# Stand dynamics and competition in a mixed forest at the northern distribution limit of evergreen hardwood species

**DOI:** 10.1002/ece3.4592

**Published:** 2018-10-18

**Authors:** Koichi Takahashi, Yoshifumi Ikeyama, Isao Okuhara

**Affiliations:** ^1^ Department of Biology Faculty of Science Shinshu University Matsumoto Japan; ^2^ Institute of Mountain Science Shinshu University Matsumoto Japan; ^3^ Graduate School of Science and Technology Shinshu University Matsumoto Japan

**Keywords:** coexistence, competition, disturbance, ecotone, matrix model, permanent plot, population growth rate, spatial distribution

## Abstract

Tree species of three growth forms (evergreen conifers, deciduous hardwoods, and evergreen hardwoods) codominate at the northern distribution limit of evergreen hardwoods in central Japan. This study examined the stand dynamics and competition during 13 years at a single plot to reveal how three growth forms codominate at the ecotone. Species were characterized as large DBH and low tree density for evergreen conifers, and conversely for evergreen hardwoods. Total basal area increased during the examined period, accompanied with the reduction in tree density (i.e., mortality exceeded the recruitment rate). Mortality increased with time especially for small trees of deciduous hardwoods. The effect of competition among the three growth forms on tree growth was not detected. Species were classified into two axes. Ingrowth and recruitment rates of large evergreen conifers were lower than those of small evergreen hardwoods. The population growth rate was lower in species with greater mortality within each growth form. Deciduous hardwoods showed the highest mortality and lowest population growth rates among the three growth forms. Although the tree‐ring analysis revealed that disturbances occurred to some extent, the current disturbance regime would not trigger the regeneration of deciduous hardwoods. This study suggests that negative relations of maximum DBH with ingrowth and recruitment rates contribute to codominance of evergreen conifers and evergreen hardwoods, and more frequent or larger disturbances than at present are necessary for regeneration of deciduous hardwoods.

## 1 INTRODUCTION

The dominant growth form of tree species changes with increasing latitude from evergreen hardwoods in tropical rain forests to deciduous hardwoods in temperate forests and to evergreen conifers in boreal forests. Temperate conifers (such as Cupressaceae and Sciadopityaceae) distribute in the temperate zone (Horikawa, 1972). Species composition of vegetation steeply changes at certain sites (i.e., ecotone), rather than gently changes along environmental gradients, such as latitude and elevation (Goldblum & Rigg, 2010; Martin, Canham, & Kobe, 2010; Takahashi, 2003). A vegetation ecotone is the boundary between adjacent communities, and so includes the range limit of species distribution. Ecotone is important for understanding the formation of vegetation distribution and maintenance of species diversity, because species with different distribution ranges (or different growth forms such as deciduous and evergreen trees) codominate at the same ecotone, for example, northern‐upper and southern‐lower distribution limits (Messaoud, Bergeron, & Leduc, 2007; Takahashi, 2014).

It is no doubt that disturbance is important to maintain species diversity (Pickett, 1980). Especially, in forest ecosystems with developed stratification, seedling establishment and population growth rates of shade‐intolerant species gradually decrease with stand development (Takahashi, 2010a; Takahashi, Yogo, & Ishibashi, 2006). However, shade‐intolerant species can dominate immediately from newly established seedlings after large disturbances because of high growth rates in bright conditions. Thus, large disturbances interrupt competitive exclusion of shade‐intolerant species by shade‐tolerant species, which maintains species diversity (Firm, Nagel, & Diaci, 2009; Lusk & Ogden, 1992).

A large disturbance sometimes does not occur more than a millennium (Lorimer, 1977). In equilibrium conditions without large disturbances, species diversity is maintained by species differences in regeneration traits (microhabitat preference, recruitment, growth and longevity, etc.) (Easdale, Healey, Grau, & Malizia, 2007; Shmida & Ellner, 1984; Takahashi, 2017). For example, maximum plant size relates to interspecific variations of regeneration traits in forests. Although taller trees are unquestionably advantageous for competition for light (Weiner, 1990), higher carbon allocation to the trunk is required for taller tree species to increase the trunk height. Therefore, reproduction of taller tree species starts later (Thomas, 1996), which may decrease lifetime resource allocation for reproduction. The trade off between maximum plant size and reproduction contributes to maintenance of species diversity in forest ecosystems (Kohyama, 1993).

Intraspecific and interspecific competition also relates to maintenance of species diversity. Species coexist if intraspecific competition is stronger than interspecific competition, according to the classical Lotka–Volterra competition equation (Begon, Harper, & Townsent, 1996). Especially, spatial distribution of individuals largely affects intraspecific and interspecific competition of sessile plants (Duncan, 1991; Takahashi & Kohyama, 1999; Takahashi, Mitsuishi, Uemura, Suzuki, & Hara, 2003). Therefore, the maintenance of species diversity relates to disturbance, tree competition and species differences in regeneration traits. However, how populations of some growth forms are maintained at a latitudinal ecotone is rarely investigated from the viewpoints of tree competition and species differences in regeneration traits.

Central Japan is the vegetation ecotone between southern evergreen hardwood forests and northern deciduous hardwood forests, with a mixture of temperate evergreen conifers in some areas (Horikawa, 1972). Therefore, central Japan is an ideal site to analyze how growth forms evergreen conifers, deciduous, and evergreen hardwoods codominate through tree competition and species differences in regeneration traits. This study aimed to show regeneration dynamics in a mixed stand of the three growth forms at the vegetation ecotone in central Japan. First, we describe the species composition, size structure, and stand structural changes during 13 years. Second, we examine demographic rates (mortality, growth, and recruitment rates), population growth rates, spatial patterns of trees, tree competition, and growth releases from suppression due to disturbances by using 13‐year observation data. Specifically, we attempt to answer the following questions.

1. Has the stand experienced exogenous disturbances?
2. Are size structure and population growth rates stable for each species by balancing the recruitment rate and mortality?
3. Are recruitment rates lower in species of larger maximum size?
4. Is competition among the three growth forms an important factor for their growth through spatial distribution?

## 2 MATERIALS AND METHODS

### 2.1 Study site

This study was conducted at 400~500 m above sea level (a.s.l.) in an old‐growth stand on the northern slope in the Shizumo Forest Reserve (101.4 ha) on Mount Shizumo (767 m a.s.l., N35°35′, E137°34′), central Japan. Although this forest reserve was established in 1962, there was no anthropogenic disturbance at this stand before 1962 (Yokouchi, Iinuma, & Yokouchi, 1963). A few trees were over 250 years old. The site was the unstable granite substrate on the steep slope (about 30°) (Yokouchi et al., 1963).

The mean annual temperature was estimated as 11.9°C at this study site from temperatures recorded at Nagiso Weather Station (560 m a.s.l., N35°36.6′, E137°37.2′, about 5 km from the study area) between 1981 and 2010 (Japan Meteorological Agency, http://www.jma.go.jp/jma/index.html [last accessed on 30 March 2017]), using the standard lapse rate (−0.6°C/+100 m). The mean temperatures of August and January were 24.0 and −0.1°C, respectively. The annual mean precipitation was 2,413 mm between 1981 and 2010 at Nagiso, with most precipitation in summer.

Relationships between plant distribution and climate are often represented by the warmth index (WI) and cold index (CI) (Kira, 1949). WI is calculated as Σ (*m*_*t*_ − 5), where *m*_*t*_ is the monthly mean temperature greater than 5°C. CI is calculated as −Σ (5 − *m*_*t*_), where *m*_*t*_ is the monthly mean temperature lower than 5°C. Generally, the distribution range of evergreen hardwood forests is WI 85~180°C months and CI > −10°C months. Evergreen hardwood forests are not distributed at CI < −10°C months because of the cold, even if the WI during the growth period is high enough for growth. The WI was 94.6°C months at the study site, and the CI was −11.9°C months. Thus, the thermal condition of the study site is a climate range limit for evergreen hardwoods (Takahashi & Okuhara, 2012).

### 2.2 Field methods

#### 2.2.1 Plot survey

A 50 × 100 m plot was established in the Shizumo Forest Reserve in 2002 and was divided into 10 × 10 m quadrats. All trees ≥2.0 cm diameter at breast height (DBH) were tagged, species were identified, and their girth at breast height and coordinates of spatial position were measured in 2002. These censuses were also done in 2007, 2012, and 2015. Dead trees and newly recruited trees growing to ≥2.0 cm DBH were recorded in each census. Dead trees were classified as standing dead, stem broken, and uprooted. Standing dead trees were assumed to have died of senescence or suppression, and stem broken and uprooted trees were assumed to have died of disturbances (Nakashizuka et al., 1992; Takahashi, 2010b). Nomenclature was followed by Shimizu (1997).

#### 2.2.2 Wood core sampling

To investigate growth releases from suppression by disturbances, wood cores were sampled from two species from each of the three growth forms in 2007. Sampled species were evergreen hardwoods *Cleyera japonica* and *Eurya japonica*, deciduous hardwoods *Fagus japonica* and *Magnolia hypoleuca* and evergreen conifers *Chamaecyparis obtusa* and *Abies firma*. These species were chosen because their maximum DBHs were greater than those of the other species in each growth form. At least 20 trees were cored at 1.3 m trunk height for each species, with two cores from each tree. All cores were dried, mounted, sanded, and then the tree‐ring widths were measured at precision 0.01 mm under a microscope by using a measurement stage (TA Tree‐Ring System, Velmex Inc., NY, USA).

### 2.3 Data analysis

#### 2.3.1 Ingrowth, mortality, and recruitment rates

In this study, population dynamics were examined for species with the percentage of density or total basal area to the plot total greater than 2.0% at the first census in 2002. Demographic rates of ingrowth, mortality, and recruitment were calculated for each species for 2002–2007, 2007–2012, and 2012–2015. Ingrowth rates of surviving trees were calculated by the following equation:

$$\text{Ingrowth rate}\;\left(\%\;{\text{year}}^{-1}\right)=\ln\left({\text{BA}}_{s}/{\text{BA}}_{i}\right)\times 100/t,$$

where ln is a natural logarithm, BA_*i*_ is the total basal area of trees at the initial census, and BA_*s*_ is the total basal area of surviving trees during the census period (*t* years). Mortality and recruitment rates were also calculated by the following equations (Condit, Hubbel, & Foster, 1995; Sheil & May, 1996):

$$\text{Mortality}\;\left(\%\;{\text{year}}^{-1}\right)=\ln\left(N_{i}/N_{s}\right)\times 100/t,$$

$$\text{Recruitment rate}\;\left(\%\;{\text{year}}^{-1}\right)=\ln\left(N_{f}/N_{s}\right)\times 100/t,$$

where *N*_*i*_ is the initial number of trees at the initial census, *N*_*s*_ is the number of surviving trees during the census period (*t* years) and *N*_*f*_ is *N*_*s*_ plus the number of recruits growing to ≥2.0 cm DBH during the census period (*t* years).

Mortality was also calculated for arbitrary DBH classes 2.0–3.9, 4.0–7.9, 8.0–15.9, 16.0–31.9, and ≥32 cm for 2002–2007, 2007–2012, and 2012–2015 for pooled data of each growth form to examine how mortality changed with tree size during 13 years (2002–2015). The number of larger trees was less, so we adopted broader DBH ranges for larger DBH classes to ensure the number of trees at each DBH class. The initial number in each size class was recalculated for the second and third periods. Recruitment trees growing to ≥2.0 cm DBH by 2007 and 2012 were included in the initial numbers of trees in the second and third periods, respectively. The number of dead trees and trees growing to the next size class were also considered for recalculation of the initial number of each size class. Calculation of the mortality for each DBH class of each census period was not done for each species because of insufficient sample size. A 95% confidence interval of mortality and recruitment rates were generated using a 1,000 iteration bootstrap technique (Crowley, 1992).

#### 2.3.2 Detection of release events of tree growth

Although we sampled two cores from each tree, tree‐ring widths were highly positively correlated between two cores of each tree. Therefore, only one core with the longer chronology was used for each tree to detect the release events of growth. The release events of growth were detected by using the R package TRADER (Altman, Fibich, Dolezal, & Aakala, 2014) that used the method of Nowacki and Abrams (1997). In this method, the average tree‐ring width over the immediately preceding 10 years, *M*_1_ (including the target year), and the average tree‐ring width over the immediately subsequent 10 years, *M*_2_ (excluding the target year), are computed, and the percentage growth change is obtained as [(*M*_2_ − *M*_1_)/*M*_1_] × 100. The minimum thresholds applied for releases are typically a 25% and a 50% growth change for moderate and major releases, respectively (Altman et al., 2014). We counted the number of trees with growth change ≥25% (i.e., moderate plus major releases) of each year for each species. The results of growth releases were summarized to 5‐year intervals, which minimized the bias caused by measurement errors and the lag of tree response to disturbances (Altman et al., 2014; Lorimer & Frelich, 1989).

#### 2.3.3 Spatial associations among the three growth forms

We analyzed the spatial association between overstory trees (DBH ≥ 10.0 cm) and understory trees (DBH 2.0–9.9 cm) of the three growth forms by the *L*_12_ (*r*)‐function (Lotwick & Silverman, 1982), an extension of Ripley's *K* (*r*)‐function (Ripley, 1977). The *L*_12_ (*r*)‐function is based on tree‐to‐tree distances (*r*). A value of the *L*_12_ (*r*)‐function equal to 0 indicates a mutually independent distribution, *L*_12_ (*r*) > 0 for a positive association, and *L*_12_ (*r*) < 0 for a negative association. Spatial association between two populations is tested conditionally against the spatial pattern of each population, classically by shifting points of a population by a random vector over a torus, while the pattern of points of the other population is unchanged (Lotwick & Silverman, 1982). The Monte Carlo simulation is used to assess the significance of deviation from the complete spatial randomness assumption, generating many random spatial patterns that provide a 99% confidence envelope. Values of the *L*_12_ (*r*)‐function higher than, equal to and lower than the 99% confidence envelopes show a positive association, a mutually independent distribution and a negative association, respectively (Lingua, Cherubini, Motta, & Nola, 2008).

In this study, we calculated the *L*_12_ (*r*)‐function for the nine combinations between understory trees (DBH 2.0–9.9 cm) and overstory trees (DBH ≥ 10.0 cm) of the three growth forms using 1,000 simulations for each. The *L*_12_ (*r*)‐function was calculated between 1 m and 10 m for distance *r* by using the package ads (Pélissier & Goreaud, 2015) for free statistical software R (R Core Team, 2017). Although we calculated the *L*_12_ (*r*)‐function for data of both 2002 and 2015, the results were very similar between the two years. Therefore, the 2002 results only are shown in this study.

#### 2.3.4 Tree competition among tree growth forms

The absolute diameter growth rate (ADGR) of trees was analyzed in relation to tree competition among the three growth forms. The ADGR of each tree is reduced by local crowding of neighboring trees. Many forest plot studies often defined neighborhood area as a 10 × 10 m quadrat in which the target tree is located to analyze tree competition (Kohyama, 1992, 1993; Nakashizuka & Kohyama, 1995; Takahashi & Kohyama, 1999). Thus, this study applied the definition of the neighborhood area as a 10 × 10 m quadrat as used in many previous studies.

Each plant acquires soil water and nutrients in proportion to its size (i.e., two‐sided competition or symmetric competition). On the contrary, in terms of competition for light, larger plants acquire a greater light resource disproportionately to their size (i.e., one‐sided competition or asymmetric competition) (Hara, 1992; Kikuzawa, 1999; Takahashi, Uemura, Suzuki, & Hara, 2003; Weiner, 1990). This study examined these two modes of competition. For two‐sided competition, all trees (DBH ≥ 2.0 cm) within the neighborhood area were treated as neighboring trees. For one‐sided competition, only trees with DBH larger than the target tree were treated as neighboring trees. Local crowding was calculated for each target tree as the sum of the basal area of each growth form of its neighbors.

A generalized linear mixed model was used to analyze the effects of local crowding of the three growth forms on the ADGR (cm/year) of each growth form. The ADGR often positively correlates with DBH and differs among tree species, even trees with the same DBH (Easdale, Allen, Peltzer, & Hurst, 2012; Hara, Kimura, & Kikuzawa, 1991; Takahashi, 2010b; Takahashi et al., 2006). The fitness (*R* ^2^) of the linear regression (ADGR as a dependent variable) was compared between DBH and ln‐transformed DBH as independent variables before the competition analysis. Because *R* ^2^ values were not so different (about 0.01) between the two regression models for each growth form, this study used DBH as an independent variable for the competition model. The model including all variables is:

$$\begin{array}{cc} \text{ADGR}= & a_{0}+a_{1}\;\text{Species}+a_{2}\;\text{DBH}+a_{3}\;\Sigma \;{\text{BA}}_{\mathrm{EC}1}+a_{4}\;\Sigma \;{\text{BA}}_{\mathrm{DH}1}+a_{5}\;\Sigma \;{\text{BA}}_{\mathrm{EH}1}+a_{6}\;\Sigma \;{\text{BA}}_{\mathrm{EC}2}\\ & +a_{7}\;\Sigma \;{\text{BA}}_{\mathrm{DH}2}+a_{8}\;\Sigma \;{\text{BA}}_{\mathrm{EH}2}, \end{array}$$

where *a*_0_ ~ *a*_8_ are coefficients. Species were treated as a categorical variable, and the other variables were continuous variables. DBH is the initial DBH (cm) of the measurement period. ∑BA_EC*i*_, ∑BA_DH*i*_, and ∑BA_EH*i*_ (cm^2^/m^2^) are total basal areas of evergreen coniferous neighbors, deciduous hardwood neighbors, and evergreen hardwood neighbors, respectively. A subscript *i* of ∑BA_EC*i*_, ∑BA_DH*i*_, and ∑BA_EH*i*_ represents one‐sided competition (*i *=* *1) or two‐sided competition (*i *=* *2). The ADGR of each target tree and local crowding were calculated for 2002–2007, 2007–2012, and 2012–2015 and were used in the model calculation. An individual tree was treated as a random effect.

Regression models were developed for all possible combinations of explanatory variables without interactions, yielding a total of 256 possible models. The model with the lowest Akaike information criteria was selected as being the best. The R package glmmML was used for the analysis.

#### 2.3.5 Population growth rate

The population growth rate of each dominant species was calculated by using a stage‐classified matrix model (Caswell, 2001). Population dynamics is often a density‐dependent process, that is, life‐history demographic rates, such as growth, survival and fertility, decrease with increasing population density or local crowding (Bai et al., 2012; Brandeis, Newton, & Cole, 2001; Castagneri, Lingua, Vacchiano, Nola, & Motta, 2010; Takahashi, 1996, 2010b; Zhang, Huang, & He, 2015). However, the size of the study plot was not large, so we could not analyze the density dependency for life‐history demographic rates of each dominant tree species. Therefore, we treated the demographic rates of fertility, growth, and survival as constants without the density dependency. [Link], [Link] describe the parameter estimation and population projection matrices of species and the model calculation.

A principal component analysis (PCA) was done to show regeneration traits of each species of the three growth forms. Explanatory variables used in the analysis were as follows: maximum DBH, recruitment, mortality, ingrowth, and population growth rates. All statistical analyses were done using the free software R (var. 3.3.3) (R Core Team, 2017).

## 3 RESULTS

### 3.1 Species composition and size structure

The initial tree density (DBH ≥ 2 cm) was 1804 trees/ha, and the total basal area was 35 m^2^/ha (Table 1). The numbers of species were 6, 30, and 10 species for evergreen conifers, deciduous hardwoods, and evergreen hardwoods, respectively. The number of species ≥2% in density or the total basal area of the plot total was five species for each of evergreen conifers and evergreen hardwoods, and six species for deciduous hardwoods. Although the total species number of deciduous hardwoods was much more than evergreen conifers and evergreen hardwoods, the density of many deciduous hardwood species was low. Most dominant species were *Abies firma* and *Chamaecyparis obtusa* for evergreen conifers, *Fagus japonica* and *Sapium japonicum* for deciduous hardwoods, and *Illicium anisatum* and *Eurya japonica* for evergreen hardwoods (Table 1). Conifer species other than the five species was only *Cephalotaxus harringtonia* (Table 1).

**Table 1:** Species composition (DBH ≥ 2.0 cm) at the study plot in a conifer‐hardwood mixed forest at the northern distribution limit of evergreen hardwood species in central Japan. Species greater than 2% relative density or relative basal area in 2002 are listed and are arranged by descending order of basal area for each growth form

Growth form and species	2002		2015		Observed max. DBH	Mortality	Recruit	Ingrowth
	Density	Basal area	Density	Basal area				
	(ha^−1^)	(m^2^/ha)	(ha^−1^)	(m^2^/ha)	(cm)	(% year^−1^)	(% year^−1^)	(% year^−1^)
Evergreen conifers								
*Abies firma*	98	7.44	82	7.73	108.4	1.956	0.585	1.425
*Chamaecyparis obtusa*	72	6.88	70	6.69	105.2	0.669	0.453	1.279
*Torreya nucifera*	34	2.33	30	2.76	109.1	1.494	0.531	1.341
*Tsuga sieboldii*	18	2.24	20	2.44	103.3	0.906	1.716	0.994
*Sciadopitys verticillata*	32	1.94	30	2.45	114.2	0.496	0.000	1.814
Others (*Cephalotaxus harringtonia*)	8	<0.01	6	<0.01	4.0	5.332	3.119	1.475
Deciduous hardwoods								
*Fagus japonica*	150	3.97	100	3.67	61.7	3.433	0.314	2.198
*Carpinus japonica*	30	1.20	16	1.16	46.0	5.863	1.027	2.758
*Carpinus laxiflora*	28	1.18	24	1.44	53.0	1.186	0.000	1.553
*Acer sieboldianum*	32	0.77	30	0.89	39.9	0.496	0.000	1.056
*Sapium japonicum*	156	0.66	132	0.86	19.4	1.766	0.481	2.623
*Callicarpa japonica*	42	0.04	16	0.05	12.1	14.969	7.545	1.993
Others (24 species)	216	2.25	168	3.18		4.025	2.092	2.848
Evergreen hardwoods								
*Illicium anisatum*	354	1.75	410	1.71	22.2	2.084	3.213	1.592
*Quercus myrsinifolia*	98	0.61	92	0.89	37.7	1.005	0.519	2.987
*Eurya japonica*	230	0.56	288	0.84	14.5	0.412	2.248	2.940
*Pieris japonica*	110	0.48	70	0.35	20.0	3.700	0.223	1.152
*Quercus salicina*	38	0.15	34	0.20	16.9	1.322	0.466	2.520
Others (5 species)	58	0.49	58	0.61		1.783	1.783	2.896
Total								
Evergreen conifers	262	20.84	238	22.07		1.344	0.605	1.363
Deciduous hardwoods	654	10.08	486	11.23		3.370	1.086	2.254
Evergreen hardwoods	888	4.03	952	4.60		1.613	2.181	2.263
Total	1,804	34.95	1,676	37.90		2.162	1.614	1.714

**Figure 1:**
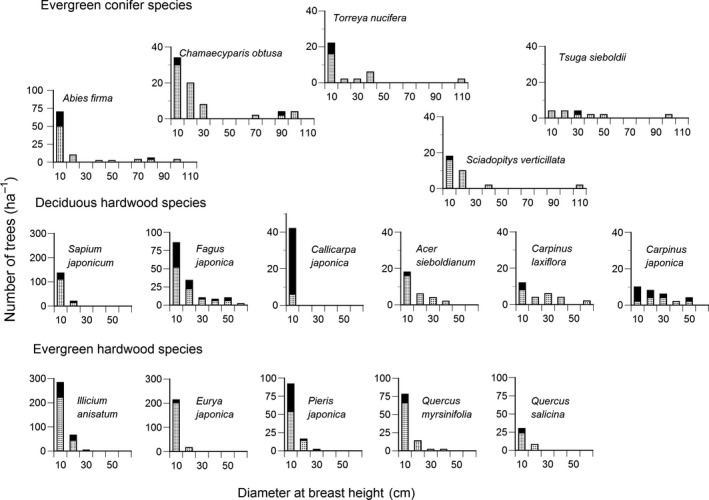
Frequency distribution of diameter at breast height at the initial census (2002) for five evergreen conifer species, six deciduous hardwood species, and five evergreen hardwood species. Dark shade is the number of individuals that died by the final census (2015). Note that the scale of vertical axis differs among the graphs

The DBH frequency distribution of each species other than *Tsuga sieboldii* showed a reverse J‐shaped pattern with many small trees (Figure 1). *Tsuga sielboldii* showed a flat‐shaped DBH frequency distribution. The tree density of the five evergreen conifer species was low, and middle‐sized trees were often absent in their DBH frequency distributions. The maximum DBH of the conifer species exceeded 100 cm (Figure 1), and their total basal area occupied 60% of the plot total (Table 1). The total density of evergreen hardwoods occupied 49% of the plot total, but most evergreen hardwoods were small trees (Figure 1) and their total basal area was only 12% of the plot total (Table 1). The maximum DBH of the evergreen hardwood species was smaller than 40 cm. Although *Quercus salicina* was a tall tree species, the maximum DBH was only 17 cm at this plot. The maximum DBH of the deciduous hardwoods varied between 12.1 cm (*Calliparpa japonica*) and 61.7 cm (*Fagus japonica*).

### 3.2 Mortality, recruitment, and ingrowth rates

**Figure 2:**
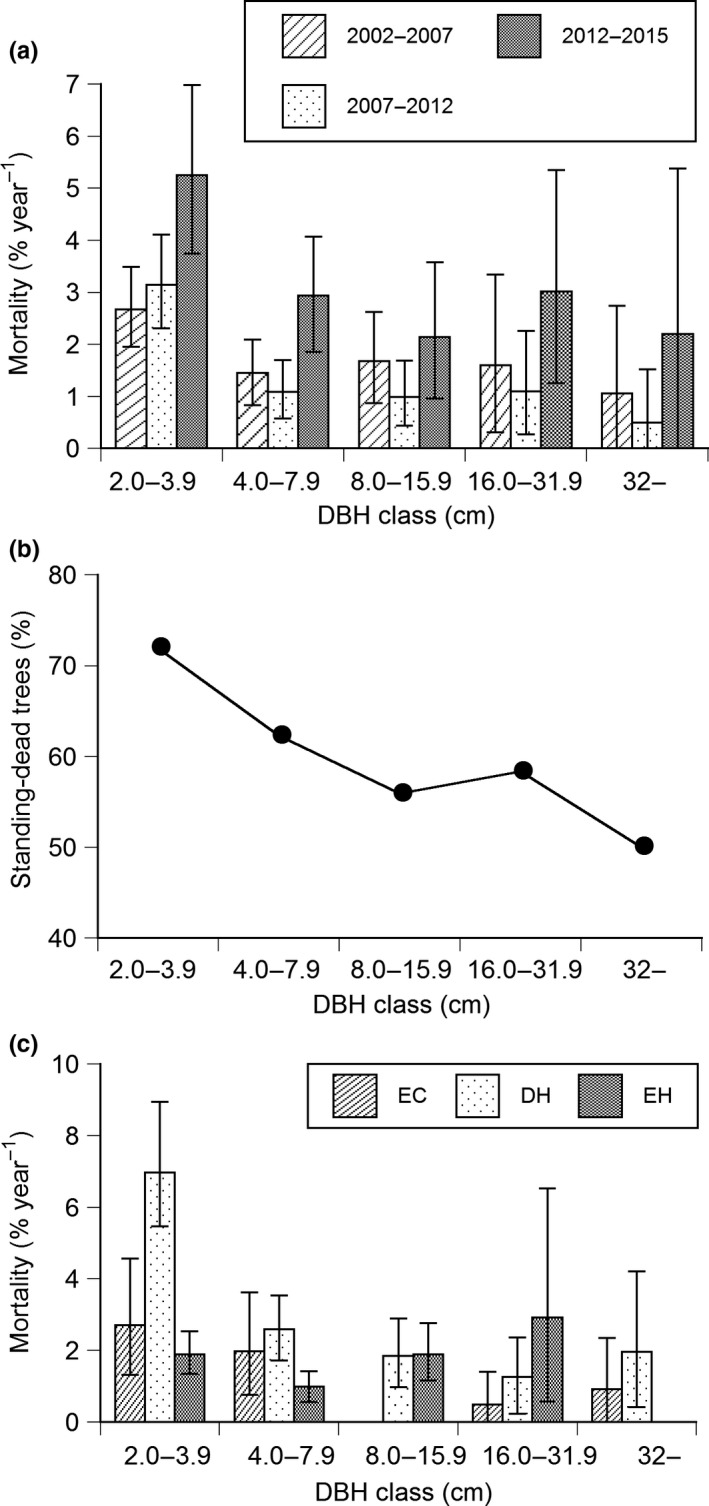
(a) Mortalities of five DBH classes for 2002–2007, 2007–2012, and 2012–2015. (b) Percentages of standing‐dead trees to total dead trees of five DBH classes for pooled data of evergreen conifers, deciduous hardwoods, and evergreen hardwoods during 2002–2015. (c) Mortalities of evergreen conifers (EC), deciduous hardwoods (DH), and evergreen hardwoods (EH) of five DBH classes during 2002–2015. (a, c) Vertical bars indicate 95% confidence intervals generated using a 1,000 iteration bootstrap technique

The total density of the plot decreased, while the total basal area increased during 13 years, 2002–2015 (Table 1). Especially, the reduction in density was conspicuous in deciduous hardwoods. By contrast, the density increased in two of the five evergreen hardwood species. Mortality was greater in smaller DBH classes during the 13 years and tended to increase in all DBH classes in the last period, 2012–2015 (Figure 2a). More dead trees showed the form of standing dead at smaller classes (Figure 2b), indicating the main cause of death for small trees was suppression. Mortality of small trees tended to be greater for deciduous hardwoods than evergreen conifers and evergreen hardwoods (Figure 2c). However, the species difference in mortality was large among six deciduous hardwood species. Mortality was high in *Callicarpa japonica* and *Carpinus japonica*, while mortality was low in *Acer sieboldianum* and *Carpinus laxiflora* (Figure 1, Table 1). The number of surviving individuals was only three individuals of *Callicarpa japonica* during 2002–2015, which possibly caused overestimation of mortality because the number of surviving individuals is the denominator of the mortality equation.

**Figure 3:**
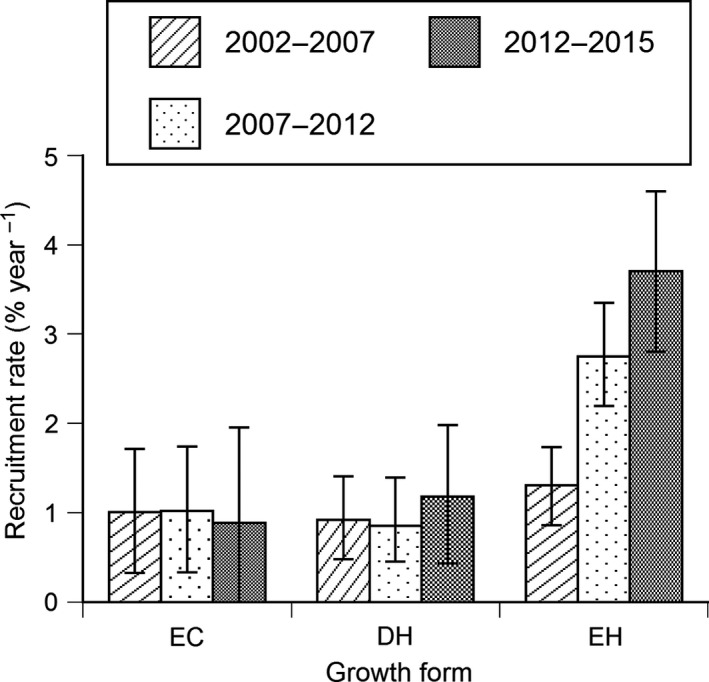
Recruitment rates of evergreen conifers (EC), deciduous hardwoods (DH) and evergreen hardwoods (EH) for 2002–2007, 2007–2012, and 2012–2015. Vertical bars indicate 95% confidence intervals generated using a 1,000 iteration bootstrap technique

Recruitment rates of trees growing to ≥2.0 cm DBH were 0.6% year^−1^ and 1.1% year^−1^ for evergreen conifers and deciduous hardwoods, respectively, during the 13 years (Table 1). The recruitment rate of evergreen hardwoods was greater than that of evergreen conifers and deciduous hardwoods, and gradually increased with time (Figure 3). The recruitment rate during the 13 years was lower than mortality for evergreen conifers and deciduous hardwoods, while the recruitment rate was greater than mortality for evergreen hardwoods (Table 1). Like mortality, the species difference in recruitment rate was large for each growth form, especially for deciduous hardwoods. The recruitment rate was highest for *Calliparpa japonica* among the six deciduous hardwood species, while no recruitment was observed for *Carpinus laxiflora* and *Acer sieboldianum*. However, like mortality of *Calliparpa japonica*, the recruitment rate of this species was possibly overestimated because the number of surviving individuals that is the denominator of the recruitment equation was only three individuals. The recruitment rate showed no significant correlation with mortality (*R *=* *0.160, *n *=* *16) and with maximum DBH (*R *= −0.370, *n *=* *16) for 16 species of the three growth forms.

The ingrowth rate negatively correlated with the maximum DBH of the 16 species of the three growth forms (*R *= −0.501, *p *<* *0.05, *n *=* *16). Therefore, ingrowth rates tended to be lower for evergreen conifers with a large maximum DBH, and higher for evergreen hardwoods with a small maximum DBH (Table 1).

### 3.3 Growth releases in the past

**Figure 4:**
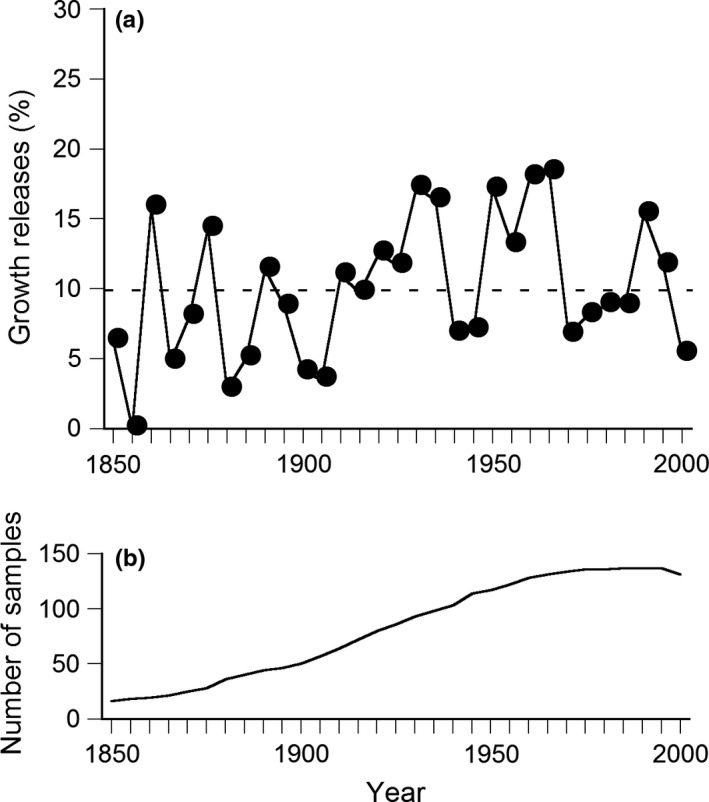
(a) Percentage of trees that showed growth releases (% per 5 years) for six tree species (two evergreen conifer species, two evergreen hardwood species, and two deciduous hardwood species). A horizontal broken line indicates 10% of growth releases. (b) The number of samples in each year

Of the six species examined, more than ten individuals were older than 100 years for conifers *Abies firma* and *Chamaecyparis obtusa*, while many individuals of deciduous hardwoods and evergreen hardwoods were younger than 100 years (Appendix S3). The percentage of trees that showed growth releases was greater than 10% per five years during 1916–1935, 1946–1965, and 1986–1995 at intervals of about 30 years after 1900 the number of samples exceeded 50 (Figure 4).

### 3.4 Spatial association and competition among the three growth forms

**Figure 5:**
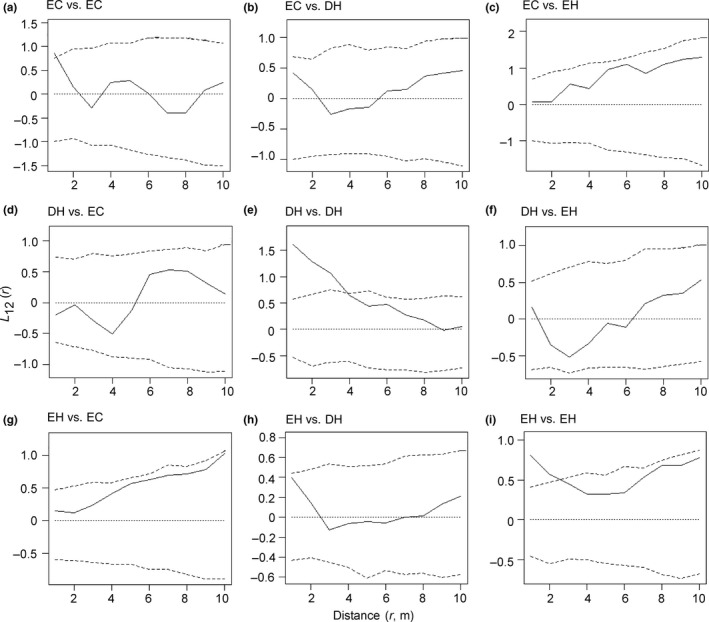
The *L*_12_ (*r*)‐function (solid line) between understory trees (DBH 2.0–9.9 cm) and overstory trees (DBH ≥ 10.0 cm) of evergreen conifers (EC), deciduous hardwoods (DH), and evergreen hardwoods (EH) in 2002, with a 99% local confidence envelope (broken lines) for a mutually independent distribution, generated by 1,000 Monte Carlo simulations. The combination of two growth forms is written as understory trees vs overstory trees in each graph, that is, (a–c) for understory trees of evergreen conifers, (d–f) for understory trees for deciduous hardwoods and (g–i) for understory trees of evergreen hardwoods. Note that the scale of vertical axis differs among the graphs

Although the number of understory trees (DBH 2.0–9.9 cm) was fewer for evergreen conifers (Appendix S4), their spatial distribution was independent of overstory trees of the three growth forms (Figure 5a–c). The spatial distribution of understory trees of deciduous hardwoods was independent of overstory trees of evergreen conifers and evergreen hardwoods (Figure 5d,f), but positively correlated with overstory trees of deciduous hardwoods in the distance <4 m (Figure 5e). Many understory trees (70%) of *Fagus japonica*, a deciduous hardwood species, were sprout origin from the base of mother trunks in 2002 (Appendix S4). Positive correlations of the *L*_12_ (*r*)‐function for the distance <4 m almost disappeared in the relationship between understory trees and overstory trees of deciduous hardwoods when *Fagus japonica* was removed from the analysis (data not shown). Therefore, the positive spatial correlation between understory trees and overstory trees of deciduous hardwoods was due to sprouts of *Fagus japonica*. Although understory trees of evergreen hardwoods showed a positive correlation with overstory trees of evergreen hardwoods for the distance <2 m (Figure 5i), the spatial distribution was almost independent of overstory trees of the three growth forms (Figure 5g–i).

The effect of tree competition among the three growth forms on the ADGR was not detected for each growth form (Table 2). The ADGR of each growth form positively correlated with DBH (Table 2). No species difference in the ADGR was detected for evergreen conifers and deciduous hardwoods. Therefore, the ADGR was determined by only DBH for evergreen conifers and deciduous hardwoods, and by DBH and species for evergreen hardwoods.

**Table 2:** The ADGR of three growth forms by generalized linear mixed models based on AIC. Individual trees were treated as a random effect, and the ADGR was calculated for 2002–2007, 2007–2012, 2012–2015) for each tree

Growth form and equation	AIC	*n*
Evergreen conifers		
ADGR = 0.065 + 0.0047 DBH	−85.0	357
Deciduous hardwoods		
ADGR = 0.056 + 0.0078 DBH	−694.8	779
Evergreen hardwoods		
ADGR = 0.0037 + 0.0041 DBH + 0.061 SP_1_ + 0.041 SP_2_ + 0.094 SP_3_ + 0.068 SP_4_ + 0.097 SP_5_	−2411.4	1,242

*Notes*

ADGR: absolute diameter growth rate (cm/year), DBH: diameter at breast height (cm).

For evergreen hardwoods, the coefficient of each species is shown as the increase from the ADGR of *Pieris japonica* (i.e., the coefficient of this species is treated as zero).

SP_1_: *Quercus salicina*, SP_2_: *Illicium anisatum*, SP_3_: *Quercus myrsinifolia*, SP_4_: *Eurya japonica*, SP_5_: other evergreen hardwood species.

### 3.5 Species differences in regeneration traits

**Figure 6:**
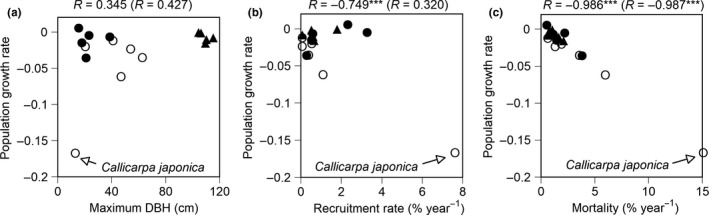
Relationships of population growth rates calculated by matrix models of 16 species, against (a) maximum DBH, (b) recruitment rate, and (c) mortality. Solid triangles, open, and solid circles indicate evergreen conifers, deciduous hardwoods, and evergreen hardwoods, respectively. Correlation coefficient (*R*) is shown in each graph; *R* values in parentheses show correlation coefficients for datasets without *Callicarpa japonica*. Asterisks indicate the statistical significance (***: *p *<* *0.001)

The population growth rate of each species was calculated by using a stage‐classified matrix model based on the observed mortality, recruitment, and growth rates. Many species showed negative population growth rates (Figure 6a). Especially, the population growth rate of *Callicarpa japonica* was much lower than for the other species. The population growth rate did not correlate with maximum DBH, while it negatively correlated with the recruitment rate (Figure 6b, *R* = −0.749, *p *<* *0.001) and mortality (Figure 6c, *R* = −0.986, *p *<* *0.001). Even if data of *Callicarpa japonica* with the extreme low population growth rate was removed, the population growth rate showed a negative correlation with mortality (Figure 6c, *R* = −0.987, *p *<* *0.001), but the significant correlation with recruitment rate disappeared (Figure 6b, *R* = 0.320).

**Figure 7:**
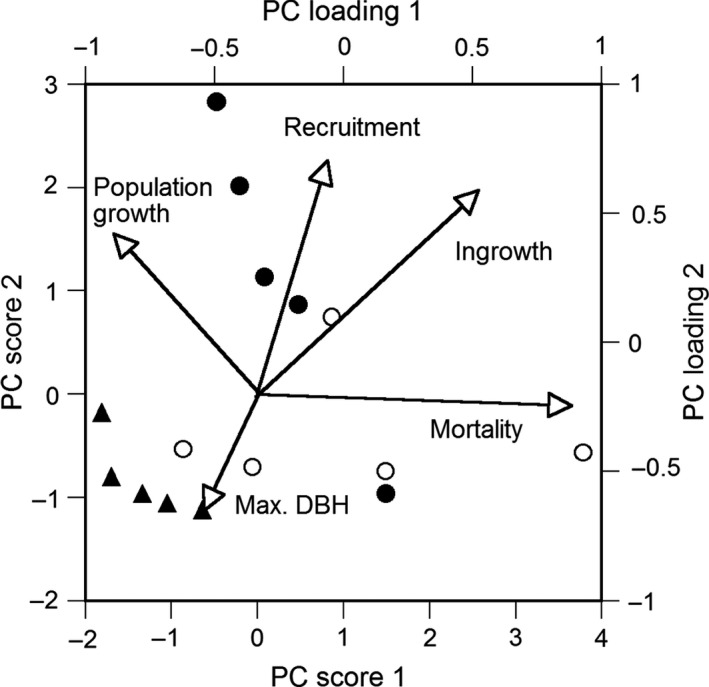
Principal component (PC) analysis of 15 species (other than *Callicarpa japonica*) of evergreen conifers (solid triangles), deciduous hardwoods (open circles), and evergreen hardwoods (solid circles) by five regeneration traits

A PCA was conducted by using maximum DBH, population growth rates and the demographic rates of mortality, recruitment, and ingrowth for species of the three growth forms. However, *Callicarpa japonica* was not used in this analysis because the overestimated recruitment rate of this species could have possibly influenced the statistical analysis (Figure 6b). Species was characterized mainly by two axes of maximum DBH and mortality (Figure 7). Ingrowth and recruitment rates were low for evergreen conifers with large maximum DBH and were high for evergreen hardwoods with small maximum DBH. The population growth rate was lower for species with higher mortality for each growth form. Deciduous hardwoods tended to have higher mortality and lower population growth rates than the two other growth forms.

## 4 DISCUSSION

### 4.1 Stand development

Many long‐term forest observational studies reported that mortality exceeds recruitment rates and that forest stands develop with reduced tree density, especially for early successional species (Abrams & Nowacki, 1992; Harcombe et al., 2002; Leak & Smith, 1996; Takahashi, 2010a; Vepakomma, Kneeshaw, & St‐Onge, 2010). This is also true in the forest stand examined in this study, that is, the total basal area increased, accompanied with reduced tree density, during 13 years. As dominant trees grow larger, there is less space for competitors to grow. Hence, smaller competitors eventually die. Stand density decreases while stand basal area increases. Disturbance occurred to some extent in the past 100 years in this study site. Probably, this forest stand would be on the way to developing from past disturbances. The mortality of deciduous hardwoods was greater than for the two evergreen growth forms, and the population growth rates of deciduous hardwoods had more negative values, indicating that regeneration of deciduous hardwoods is suggested to gradually decrease with stand development. Therefore, more frequent formation of canopy gaps or larger canopy gaps than at present is thought to be necessary for regeneration of deciduous hardwoods.

### 4.2 Maximum DBH, recruitment, and ingrowth rates

The PCA showed recruitment and ingrowth rates were greater for evergreen hardwoods with small maximum DBH than evergreen conifers with large maximum DBH. Tree species of larger maximum size should invest more carbon in growth by delaying reproduction (Thomas, 1996). In addition, the respiration rate of each individual tree is considered to disproportionately increase relative to its size because the proportion of non‐photosynthetic organs to the photosynthetic organ increases in larger plants (Givnish, 1988; Waring, 1987), that is, the carbon allocation to the growth and reproduction per photosynthetic production decreases more in larger trees. Negative correlations of recruitment rates with longevity or maximum DBH are also observed in other forests from tropical to cool‐temperate forests (Kohyama, 1993; Kohyama, Suzuki, Partomihardjo, Yamada, & Kubo, 2003; Lieberman, Lieberman, Hartshorn, & Peralta, 1985; Takahashi, Mitsuishi et al., 2003). Kohyama (1993) showed that species codominance is achieved by the trade off between the maximum size and reproduction. Therefore, the greater recruitment and ingrowth rates of evergreen hardwoods than evergreen conifers are suggested to reflect trade off relationships with maximum DBH also in this study site, which contributes to the codominance of evergreen conifers and hardwoods.

### 4.3 Regeneration of evergreen conifers

The size structure of shade‐tolerant species tends to have a reverse J‐shaped pattern because of high recruitment rates (Takahashi, 2010a; Wright, Muller‐Landau, Condit, & Hubbell, 2003). Many previous studies showed discontinuous (such as unimodal or bimodal) size and age structures of conifers in conifer‐hardwood mixed forests, indicating episodic regeneration (Hoshino, Nishimura, & Yamamoto, 2001; Nishimura et al., 2010; Stewart, 1986). Therefore, disturbance at the soil surface or canopy layer or both is thought to be necessary for regeneration of conifers (Hoshino et al., 2001; Takahashi, 2010a; Yamamoto, 1993). Although the DBH frequency distribution of the five evergreen conifer species did not show typical discontinuous patterns, such as unimodal and bimodal patterns, mid‐sized individuals were absent, indicating evergreen conifers continuously recruited to a certain extent. Our study site is a steep slope (about 30°) on the unstable granite substrate (Yokouchi et al., 1963). Therefore, small‐scale disturbances would often occur on the soil surface, which may trigger regeneration of conifers.

### 4.4 Species number of evergreen hardwoods

The total species number of deciduous hardwoods ≥2 cm DBH in the study plot was 30 species, but that of evergreen hardwoods was only 10 species. The species number of evergreen hardwoods decreases at higher elevations and latitudes (Hattori, Ishida, Kodate, & Minamiyama, 2002; Tsujino, 2016). Species more tolerant to freezing can distribute in colder areas, and generally tolerance to freezing is greater for deciduous hardwoods than evergreen hardwoods (Sakai, 1978). Takahashi and Okuhara (2012) showed that growth of evergreen hardwoods is usually restricted by winter cold in the same site as this study. The threshold temperature for tolerance to freezing is between −7°C and −18°C for evergreen hardwoods that distribute up to central Japan (Sakai, 1978). The yearly minimum temperature was estimated at between −6.1°C and −14.7°C (average −10.5°C) during 1979–2016 at this study site. The range of yearly minimum temperature almost coincides with the threshold temperature for the tolerance to freezing of evergreen hardwoods. Therefore, the species number of evergreen hardwoods is thought to be less than that of deciduous hardwoods at this forest stand at the northern distribution limit because only evergreen hardwoods with a high tolerance to freezing can grow there.

### 4.5 Regeneration of deciduous hardwoods

The mean mortality was greater and the population growth rate was lower for deciduous hardwoods than for the two evergreen growth forms. Therefore, deciduous hardwoods are thought to have regeneration niches different from the two evergreen growth forms. Especially, mortality and population growth rates of two shade‐intolerant deciduous hardwood species (*Carpinus japonica* and *Callicarpa japonica*) were greater than those of the four other shade‐tolerant deciduous hardwood species (cf. Masaki et al., 1992; Cao & Ohkubo, 1998; Tabata, Suzuki, Okumura, & Abe, 2017 for shade tolerance). Sessile plants compete with neighboring plants, so spatial distribution is an important factor for intraspecific and interspecific competition (Takahashi & Kohyama, 1999; Takahashi, Mitsuishi et al., 2003). In this study, the effect of competition among the three growth forms on their growth was not detected because of their random spatial distribution. However, the competitive interaction among growth forms would change along climatic conditions and may relate to the formation of geographical distributions of deciduous and evergreen hardwoods. Takahashi, Kobori, and Seino (2011) experimentally showed that growth rates of seedlings of both evergreen and deciduous hardwoods are greater in warmer temperature conditions, including beyond the thermal condition of the southern distribution limit of deciduous hardwood species. However, deciduous hardwoods can dominate at only the cool‐temperate zone where evergreen hardwoods cannot distribute due to the cold (Kira, 1949). That is, the result of Takahashi et al. (2011) suggests that deciduous hardwoods can distribute physiologically in the same place where evergreen hardwoods distribute, but are competitively excluded by evergreen hardwoods. Evergreen hardwoods can dominate physiologically more in warmer climatic conditions. Saplings of deciduous hardwoods distribute mainly in canopy gaps in an evergreen hardwood forest in southern Japan (Miura, Manabe, Nishimura, & Yamamoto, 2001). In deciduous hardwood forests, understory seedlings and saplings of deciduous hardwoods increase carbon gain by leafing before leaf expansion of canopy trees (Seiwa & Kikuzawa, 1996). However, the understory of evergreen hardwood forests is dark throughout a year, which may restrict seedlings and saplings of deciduous hardwoods in canopy gaps if the density of evergreen hardwoods is much greater than that of deciduous hardwoods. Therefore, actual distribution ranges of deciduous hardwoods are largely affected not only by thermal conditions, but also by competition with evergreen hardwoods.

## 5 CONCLUSION

This study showed that (a) disturbance occurred at intervals of about 30 years, (b) the size structure and population growth rate were not stable in many species (i.e., the stand was developing with the reduction in tree density), (c) recruitment and ingrowth rates of evergreen conifers of large maximum DBH were lower than those of evergreen hardwoods of small maximum DBH and (d) competition among the three growth forms was an unimportant factor for their growth because of no clear spatial correlations among the three growth forms. Probably, more frequent or large or both disturbances are necessary to maintain populations of deciduous hardwoods. Therefore, disturbance and species differences in regeneration traits (maximum size, mortality, recruitment, ingrowth rates) are important for codominance of the three growth forms. Frequency and intensity of disturbances may increase by climate change (Dale et al., 2001). Therefore, changes in disturbance and thermal conditions due to climate change are suggested to affect regeneration dynamics and species diversity of the three growth forms at the conifer‐hardwood mixed forest at the northern distribution limit of evergreen hardwoods. This study was conducted in only one plot. Stand structure and dynamics may be different depending on the location. Therefore, further research is necessary to clarify codominance mechanisms of the three growth forms in this latitudinal ecotone by examining stand structure and dynamics at many locations.

## CONFLICT OF INTEREST

None declared.

## AUTHOR CONTRIBUTION

KT designed the study. All of the authors collected the data. IO sampled wood cores and conducted the tree‐ring analysis. KT conducted the other data analyses and wrote the manuscript.

## DATA ACCESSIBILITY

Data is available via the Dryad Digital Repository.
