# Supplementary material for: Stand dynamics and competition in a mixed forest at the northern distribution limit of evergreen hardwood species
Source: Ecol Evol. 2018 Oct 18;8(22):11199–212. doi: 10.1002/ece3.4592 (PMC6262723; doi:10.1002/ece3.4592)
Supplement: Supplementary file 1 [file ECE3-8-11199-s001.docx]

**Appendix S1.** Estimation of population growth rates

Population growth rates were estimated by using a matrix model for 16 species whose percentage of density or total basal area to the plot total was greater than 2 % at the first census in 2002. A stage-classified population projection matrix is *n* (*t* + 1) = *An* (*t*), where *n* (*t*) and *n* (*t* + 1) are vectors of stage abundances at time *t* and *t* +1, respectively. A stage-classified projection matrix *A* is

$A=$ $\left(\begin{matrix} P_{1} & R_{2} & R_{3} & R_{4} & \cdots& R_{n} \\ G_{1} & P_{2} & 0 & 0 & \cdots& 0 \\ 0 & G_{2} & P_{3} & 0 & \cdots& 0 \\ 0 & 0 & G_{3} & P_{4} & \cdots& 0 \\ \vdots& \vdots& \vdots& \vdots& \ddots& \vdots\\ 0 & \cdots& \cdots& \cdots& \cdots& P_{n} \end{matrix}\right)$,

where *P_i_* is the probability of surviving and remaining in stage *i*, *G_i_* is the probability of surviving and growing from stage *i* to stage *i* + 1, and *R_i_* is the probability of recruitment of stage *i* (Caswell, 2001). The parameter estimation used for the population projection matrix for each dominant species was done by the following procedure.

1) The number of trees in 2002 was counted for each class of diameter at breast height (DBH) at 10-cm intervals, except for the smallest size class (2.0–9.9 cm).

2) The absolute diameter growth rates were calculated for each tree for census periods 2002–2007, 2007–2012 and 2012–2015 as:

ADGR = (DBH_2_ – DBH_1_) / *t*,

where ADGR is the absolute diameter growth rate during a census period (cm year^-1^), *t* is the census period (years), DBH_1_ and DBH_2_ are the DBH (cm) at the initial and final DBH (cm), respectively, during the census period. The ADGR often positively correlates with tree size in climax forests (Takahashi et al., 2003; Takahashi, 2010), and can be expressed as:

ADGR = *b*_0_ + *b*_1_ DBH,

where *b*_0_ and *b*_1_ are coefficients. Coefficient *b*_0_ and *b*_1_ were estimated by using the generalized linear mixed model (GLMM), using the data of the three census periods. Individual trees were treated as a random effect in this model. Appendix 2 lists coefficients of GLMM for the 16 tree species. The ADGRs at mid-points of all DBH classes were estimated for each species using regression equations (Appendix S2).

3) The probability (*G_i_*) of growing to the next DBH class *i* + 1 from class *i* was determined using the ADGR equations (Appendix S2). We applied the growth-frequency estimate (Gf estimate) to calculate these probabilities (Kohyama & Takada, 1998). The number of trees growing to the next DBH class *i* + 1 from DBH class *i* can be estimated by the product of the number of individuals at the size class and the ADGR (cm year^-1^) divided by the width of the size class (cm). The probability (*G_i_*) was calculated as the estimated number of trees growing to the next DBH class *i* + 1 divided by the initial number of trees at the DBH class *i*.

4) The probability (*P_i_*) of surviving and remaining in DBH class *i* is determined as 1 – *M_i_* – *G_i_*, where *M_i_* is the mortality of DBH class *i*. Mortality is a size-dependent function and often shows a U-shaped pattern with high mortality in small and large size classes (Monserud & Sterba, 1999; Takahashi & Kohyama, 1999; Takahashi, 2010). Although the mortality of the pooled data of each growth form showed a U-shaped pattern in this study, we could not analyze the size dependency of mortality for each species because of insufficient number of dead trees for the analysis of each species. Therefore, we used a constant mortality (*M_i_*), irrespective of size (DBH class *i*), for each species.

5) The recruitment rate (*R_i_*) was estimated using the number of recruits and the ingrowth of surviving trees during the 13 years from 2002 to 2015. The number of recruits into

≥ 2-cm DBH class was divided by the total ingrowth (basal area) of surviving trees within the plot during the 13 years, which is the *per-capita* recruitment rate per basal area increase for each species. The increase in basal area of DBH class *i* was calculated by using the number of trees and the ADGR of DBH class *i* for each species. Therefore, the recruitment rate (*R_i_*) was estimated by the product of the *per-capita* recruitment rate and the increase in basal area of DBH class *i*.

The matrix model was calculated to estimate the population growth rate of each species by using the PopTools (http://www.poptools.org/), an add-in for Microsoft Excel.

**References**

Caswell, H. (2001). *Matrix population models*. Sunderland: Sinauer Associates, Inc. Publishers.

Kohyama, T., & Takada, T. (1998). Recruitment rates in forest plots: Gf estimates using growth rates and size distributions. *Journal of Ecology*, *86*, 633–639.

Monserud, R.A., & Sterba, H. (1999). Modeling individual tree mortality for Austrian forest species. *Forest Ecology and Management*, *113*, 109–123.

Takahashi, K. (2010). Effects of altitude and competition on growth and mortality of the conifer *Abies sachalinensis*. *Ecological Research*, *25*, 801–812.

Takahashi, K., & Kohyama, T. (1999). Size-structure dynamics of two conifers in relation to understorey dwarf bamboo: a simulation study. *Journal of Vegetation Science*, *10*, 833–842.

Takahashi, K., Mitsuishi, D., Uemura, S., Suzuki, J., & Hara, T. (2003). Stand structure and dynamics during a 16-year period in a sub-boreal conifer-hardwood mixed forest, northern Japan. *Forest Ecology and Management*, *174*, 39–50.
