# Supplementary material for: Stand dynamics and competition in a mixed forest at the northern distribution limit of evergreen hardwood species
Source: Ecol Evol. 2018 Oct 18;8(22):11199–212. doi: 10.1002/ece3.4592 (PMC6262723; doi:10.1002/ece3.4592)

**Appendix S3.** Number of individuals examined (line) and number of individuals that showed growth changes ≥ 25% increase (dots) for each species. Two species are shown for each growth form: (a, b) evergreen conifers, (c, d) deciduous hardwoods, and (e, f) evergreen hardwoods.


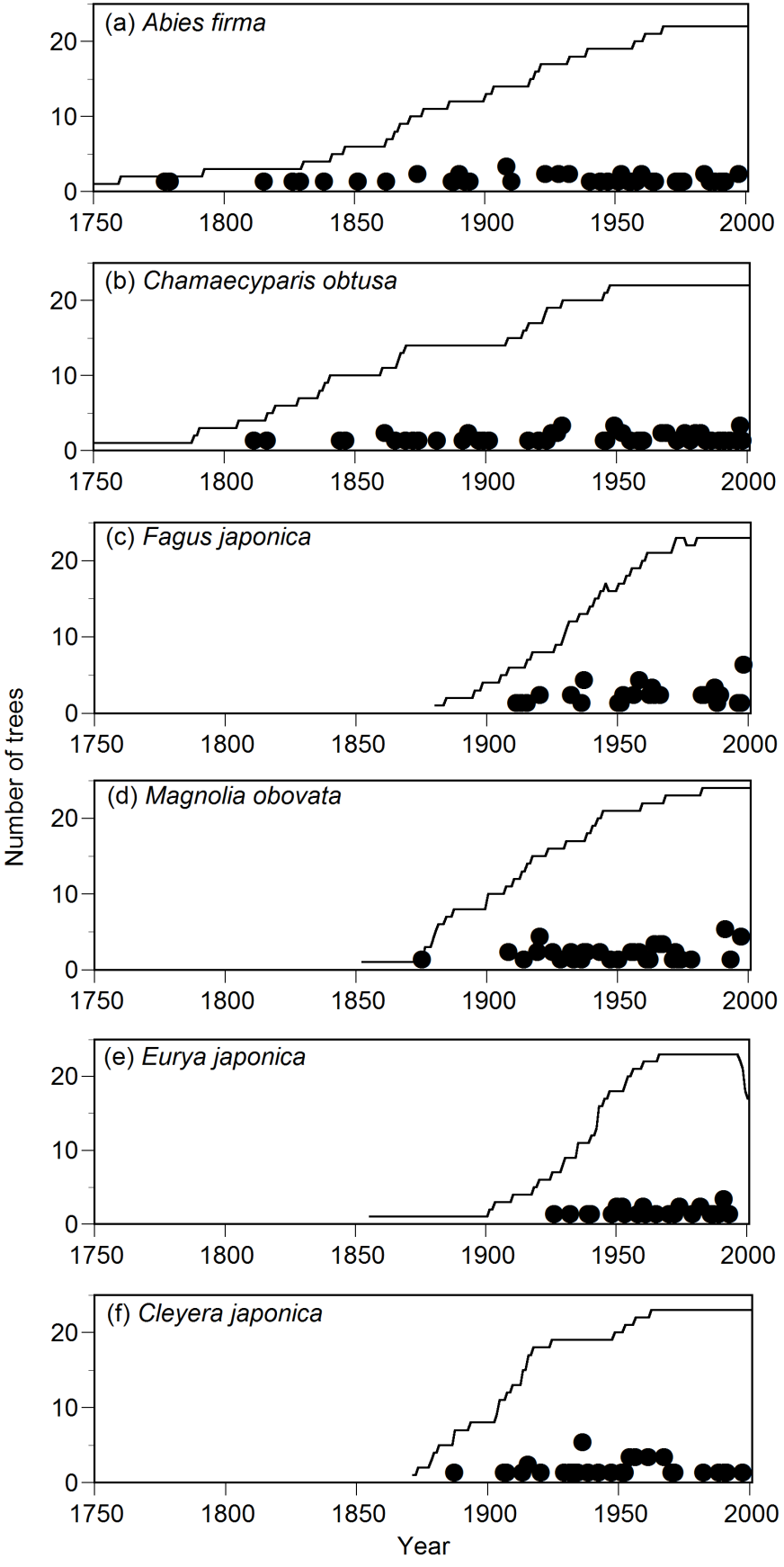

Supplement: Supplementary file 3 [file ECE3-8-11199-s003.docx]
