# Supplementary material for: Stand dynamics and competition in a mixed forest at the northern distribution limit of evergreen hardwood species
Source: Ecol Evol. 2018 Oct 18;8(22):11199–212. doi: 10.1002/ece3.4592 (PMC6262723; doi:10.1002/ece3.4592)

**Appendix S4.** Spatial distributions of understory trees (DBH 2.0‒9.9 cm, dots) and overstory trees (DBH ≥ 10.0 cm, open circles) at a plot (50 m × 100 m) for each species. *Cephalostaxus harringtonia*, an evergreen conifer species, is categorized as the other species.


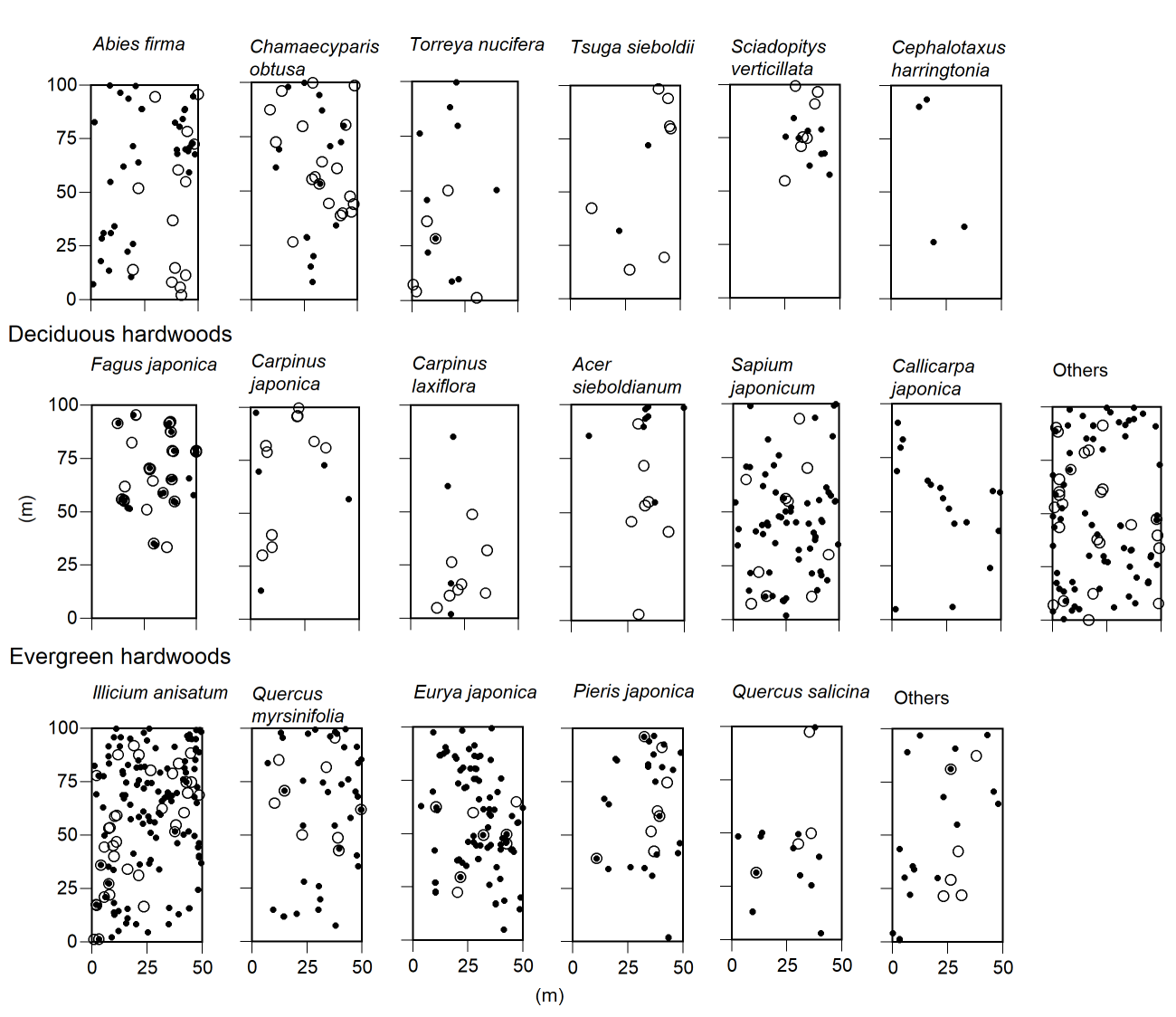

Supplement: Supplementary file 4 [file ECE3-8-11199-s004.docx]
